# Elevated lipopolysaccharide binding protein in Alzheimer’s disease patients with *APOE3/E3* but not *APOE3/E4* genotype

**DOI:** 10.3389/fneur.2024.1408220

**Published:** 2024-05-30

**Authors:** Eduardo Z. Romo, Brian V. Hong, Rishi Y. Patel, Joanne K. Agus, Danielle J. Harvey, Izumi Maezawa, Lee-Way Jin, Carlito B. Lebrilla, Angela M. Zivkovic

**Affiliations:** ^1^Department of Nutrition, University of California, Davis, Davis, CA, United States; ^2^Department of Public Health Sciences, University of California, Davis, Davis, CA, United States; ^3^Department of Pathology and Laboratory Medicine, School of Medicine, University of California, Davis, Davis, CA, United States; ^4^Department of Chemistry, University of California, Davis, Davis, CA, United States

**Keywords:** Alzheimer’s disease, *ApoE3/E3* genotype, *ApoE3/E4* genotype, lipopolysaccharide binding protein, gut permeability

## Abstract

**Introduction:**

The role of lipopolysaccharide binding protein (LBP), an inflammation marker of bacterial translocation from the gastrointestinal tract, in Alzheimer’s disease (AD) is not clearly understood.

**Methods:**

In this study the concentrations of LBP were measured in *n* = 79 individuals: 20 apolipoprotein E (APOE)3/E3 carriers with and 20 without AD dementia, and 19 *APOE3/E4* carriers with and 20 without AD dementia. LBP was found to be enriched in the 1.21–1.25 g/mL density fraction of plasma, which has previously been shown to be enriched in intestinally derived high-density lipoproteins (HDL). LBP concentrations were measured by ELISA.

**Results:**

LBP was significantly increased within the 1.21–1.25 g/mL density fraction of plasma in *APOE3/E3* AD patients compared to controls, but not *APOE3/E4* patients. LBP was positively correlated with Clinical Dementia Rating (CDR) and exhibited an inverse relationship with Verbal Memory Score (VMS).

**Discussion:**

These results underscore the potential contribution of gut permeability to bacterial toxins, measured as LBP, as an inflammatory mediator in the development of AD, particularly in individuals with the *APOE3/E3* genotype, who are genetically at 4-12-fold lower risk of AD than individuals who express *APOE4*.

## 1 Introduction

Alzheimer’s disease (AD) is a devastating neurodegenerative disease affecting >5 million Americans, and growing in prevalence (1). There is currently no cure for AD, and late-stage treatments have proven ineffective at reversing the disease (2). In addition to the well-established mechanism of neurodegeneration linked with the accumulation of amyloid beta and phosphorylated tau in senile plaques in AD (3), impaired gut barrier function, which is linked with higher circulating concentrations of immunogenic endotoxins, has also been linked with AD (4–6). Lipopolysaccharide (LPS) has been hypothesized to play a major role in the onset and progression of neurodegenerative diseases, including AD (5). In one study investigating the relationship between plasma LPS concentrations and disease severity as well as the concentrations of activated monocytes in patients with amyotrophic lateral sclerosis (ALS), a group of 18 individuals with AD was used as a control group in addition to a group of cognitively healthy age-matched individuals. Interestingly, the AD patients had even higher concentrations of LPS than the ALS patients compared to the cognitively normal controls (6). There has been surprisingly little follow-up work to these intriguing findings from 2010, despite the growing recognition that gut permeability, and its associated leakage of immunogenic LPS into the circulation is a potent driver of chronic inflammation (7) associated with a growing number of chronic conditions and diseases from Parkinson’s disease (8), to fibromyalgia (9), to high blood pressure (10), to autoimmune hepatitis (11), to osteoarthritis (12) and an array of neurological and psychiatric disorders (13).

LPS is a potent immunogenic activator of the innate immune system, entering the circulation from the intestine, mouth, or skin wounds, which is shuttled by the LPS binding protein (LBP) to innate immune cell surface receptors CD14 and TLR4, eliciting inflammatory signaling and a strong immune response (14). Perturbation of intestinal mucosal homeostasis allows immunogenic endotoxins like LPS to translocate across the gut barrier due to dysregulation of junctional complexes (15, 16). Translocation of immunogenic endotoxins across a compromised intestinal barrier can trigger a chronic inflammatory response throughout the body that has been linked to an increased risk of various chronic and acute diseases including atherosclerotic cardiovascular disease, dementia, cancer, and sepsis (6, 17–19). Importantly, LBP was recently shown to be a powerful independent predictor of AD risk in a prospective nested case–control study of 212 incident cases of AD matched with 424 controls with no dementia (20). Higher concentrations of LBP at baseline were associated with 30% higher odds of developing AD over the 12-year follow-up (20). However, the impact of apolipoprotein E (*APOE*) genotype in the relationship between LBP and AD has not been investigated. This is an important gap in current knowledge because *APOE* is the single strongest genetic risk factor for AD, with the *APOE4* allele increasing risk by 4-12-fold compared to *APOE3* (21). It is critical to understand the contribution of *APOE* genotype on the relationship between gut permeability and AD because whereas the prevalence of *APOE4* in the general population is approximately 25% in AD patients it is as high as 50–60% (22). While various functionalities related to the ApoE4 protein have been implicated in AD pathophysiology (23–25) the etiology of AD in individuals who are not carriers of *APOE4* is unclear. The hypothesis of endotoxin-mediated neurodegeneration suggests that endotoxin plays a major role in AD pathology by inducing systemic inflammation, degrading the blood brain barrier, and driving amyloid beta production and aggregation and TAU hyper-phosphorylation, as well as activating brain microglia (6). However, if *APOE* genotype is an important modifier of the effect, then studies involving human patients will need to account for *APOE* genotype to determine who is at risk for endotoxemia-induced dementia, and to plan for appropriate sample sizes for population-based studies.

High-density lipoproteins (HDL) are heterogenous nanoparticles most commonly known for their role in cholesterol homeostasis but they also play a critical role in modulating TLR4-based inflammatory responses along with LBP by trafficking, neutralizing and clearing LPS (26, 27). Recent findings indicate that HDL synthesized in the intestinal tract bind LBP to regulate LPS -mediated activation of liver inflammation (28). HDL-LBP-LPS complexes entering the fenestrated capillary and portal vein seclude LPS from hepatic TLR4+ macrophages, preventing excessive inflammation in a putative protective mechanism (28). Intestinally derived HDL make up roughly 30% of the circulating HDL pool (29). The function and composition of these intestinally derived particles has remained largely uncharacterized due to the difficulty in isolating them as a separate subclass from human plasma. However, recent evidence suggests that intestinally derived HDL are not only specialized but unique in their physicochemical characteristics. Using an *in-situ* perfusion model of mouse intestine, Yamaguchi et al. demonstrated that intestinally derived HDL are smaller and denser than liver-derived HDL (28, 30), and Andraski et al. recently also demonstrated that in humans intestinally-derived HDL are smaller and denser using a stable isotope approach (31). Thus, even though there are currently no validated methods for isolating intestinally-derived HDL particles from the circulation, nonetheless, it is possible to study the characteristics of the 1.21–1.25 g/mL density range of plasma, where these intestinally derived HDL have been found to be enriched.

The aim and scope of this study are to determine if there is a relationship between LBP found in the 1.21–1.25 g/mL density fraction of plasma in AD patients compared with age-matched cognitively normal controls, and whether this relationship is influenced by *APOE* genotype by examining the differences between individuals with the two most prevalent *APOE* genotypes, *APOE3/E3* and *APOE3/E4*. We hypothesized that LBP would be enriched in the 1.21–1.25 g/mL fraction containing dense HDL. We further hypothesized that LBP concentrations would be elevated in AD patients compared to non-demented controls, with *APOE* genotype modifying the effect, and that LBP concentrations would be associated with cognitive function.

## 2 Results

### 2.1 Demographic and clinical characteristics

Analysis of demographic and clinical characteristics revealed that controls were significantly younger compared to AD participants in the combined group [75.0 (69.0, 79.2) vs. 80.0 (76.5, 84.5) years, *p* < 0.001, Table 1]. The same pattern was observed within the *APOE3/E3* subgroup. There was no significant age difference within the *APOE3/E4* subgroup, and no significant differences were found in sex proportion, ethnicity, BMI.

**Table 1:** Participant characteristics.

	Control	AD	*p* value
n, combined	40	39	n/a
*APOE3/E3*	20	20	n/a
*APOE3/E4*	20	19	n/a
Age, years, median (25th, 75th)	75.0 (69.0, 79.2)	80.0 (76.5, 84.5)	**<0.001**
*APOE3/E3*	75.0 (68.0, 78.0)	82.0 (78.8, 87.2)	**<0.001**
*APOE3/E4*	75.5 (70.8, 82.0)	78.0 (75.0, 81.5)	0.216
Sex proportion, (male/female), combined	20:20	20:19	0.910
*APOE3/E3*	10:10	10:10	1.000
*APOE3/E4*	10:10	10:9	0.871
Ethnicity (African American/Asian/Hispanic/White), combined	10/0/10/20	6/1/9/23	0.356
*APOE3/E3*	2/0/7/11	3/1/5/11	0.809
*APOE3/E4*	8/0/3/9	3/0/4/12	0.153
BMI, kg/m^2^, median (25th, 75th)	28.6 (24.1, 31.2)	26.1 (23.4, 29.5)	0.093
*APOE3/E3*	28.2 (25.1, 30.1)	25.1 (22.9, 29.2)	0.181
*APOE3/E4*	29.6 (23.7, 32.0)	26.8 (23.4, 29.7)	0.339

Numeric variables were analyzed by using a two-tailed Wilcoxon Rank Sum Test and categorical variables were analyzed by chi-squared test.

The bold values represent statistical significance.

### 2.2 LBP in lipoprotein fractions

**Figure 1:**
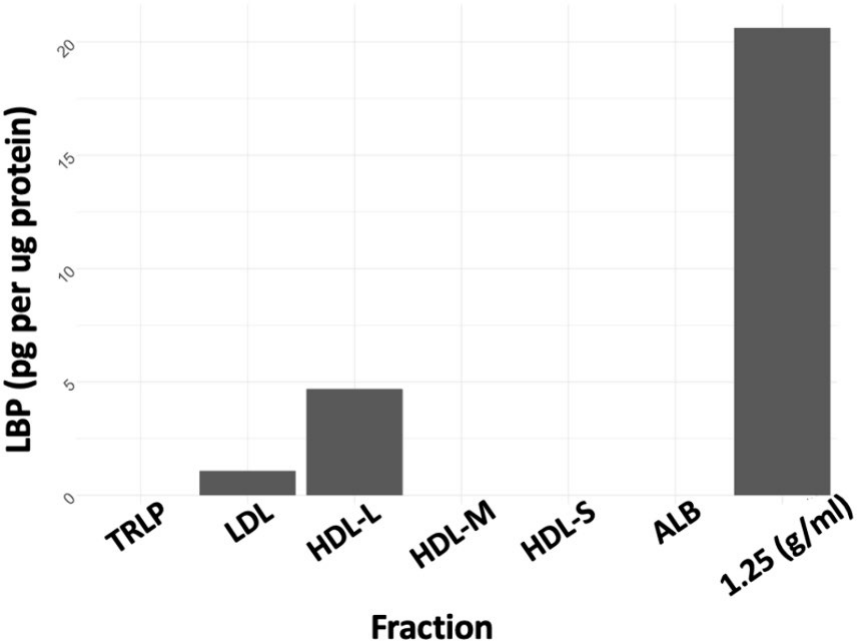
LBP as pg/μg protein. TRLP, triglyceride-rich lipoprotein; LDL, low density lipoprotein; HDL-L, large high-density lipoprotein; HDL-M, medium-high density lipoprotein; HDL-S, small high-density lipoprotein; ALB, albumin; 1.25 Density, fraction at d = 1.21–1.25 g/mL.

ELISA was conducted on all isolated plasma fractions and the concentration of LBP in each plasma fraction was determined in pg/μg protein. LBP was enriched in the 1.21–1.25 g/mL fraction (Figure 1) which was collected after ultracentrifugation at a density cutoff of 1.25 g/mL after removal of all *d* < 1.21 g/mL lipoproteins. LBP was also detected in the *d* < 1.21 g/mL large HDL fraction and LDL, however at 4–20-fold lower concentrations, respectively.

### 2.3 LBP in AD patients vs. controls

**Figure 2:**
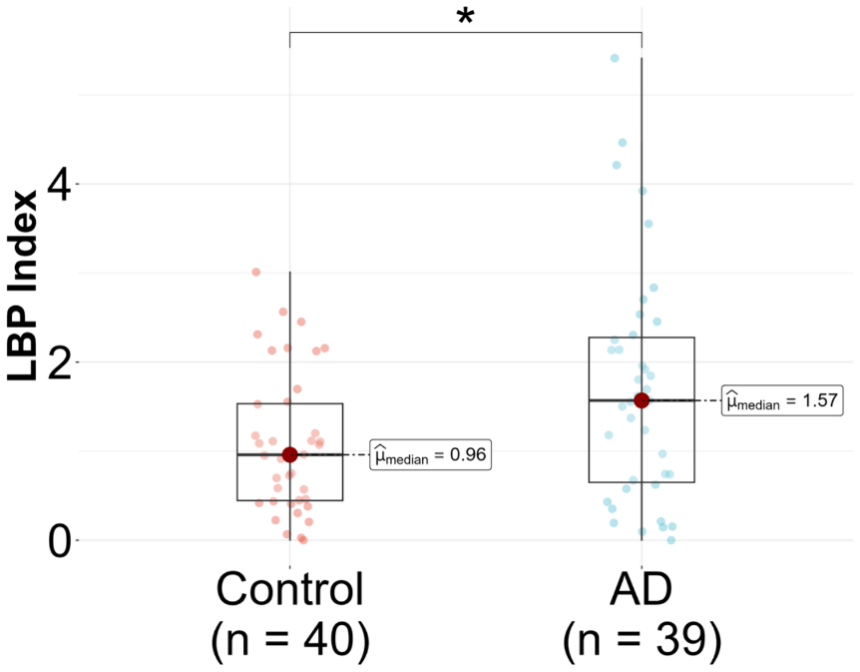
Lipopolysaccharide binding protein (LBP) in Alzheimer’s disease (AD) patients compared to controls. LBP was measured in *n* = 79 participants, 39 AD and 40 controls. Analysis was done in R utilizing a logistic regression model.

In the unadjusted logistic regression model, a statistically significant positive association was observed between the LBP index and AD diagnosis. Specifically, for each incremental unit increase in the LBP index, there was a significant increase in the likelihood of being categorized into the AD group (*β* = 0.57, *p* = 0.018) (Figure 2 and Table 2). A significant difference in the median LBP index values was also noted between the control group (0.96, 25th-75th percentile: [0.45, 1.53]) and the AD group (1.57, 25th-75th percentile: [0.65, 2.28]), indicating an elevation of the LBP index in the AD group. However, after adjusting for age, BMI, *APOE* genotype, history of diabetes, history of hypercholesterolemia, and history of hypertension, the statistical significance of the LBP index diminished (*β* = 0.39, *p* = 0.147) (Figure 2 and Table 2). The regression analysis revealed consistent relationships between LBP and AD severity measures across both sexes. No significant modulating effect of sex was observed in either the *APOE3/E3* or *APOE3/E4* genotype groups (Table 2). The lack of sex differences suggests that the findings and interpretations of the regression analysis are not significantly influenced by sex in this study.

**Table 2:** LBP index in controls and Alzheimer’s disease dementia patients.

	Result by study group		Unadjusted model		Adjusted model	
Outcome	Control (*N* = 40)	AD (*N* = 39)	Β (95% CI)	*p* value	Β (95% CI)	*p* value
LBP index ^ab^						
*APOE3/E3*+ *APOE3/E4*	0.96 [0.45, 1.53]	1.57 [0.65, 2.28]	0.57 (0.13, 1.08)	**0.018**	0.39 (−0.11, 0.96)	0.147
*APOE3/E3* ^c^	0.82 [0.43, 1.11]	1.83 [1.17, 2.58]	1.3 (0.47, 2.41)	**0.008**	2.69 (0.56, 6.19)	**0.049**
*APOE3/E4* ^c^	1.10 [0.58, 2.13]	1.18 [0.32, 2.05]	0.07 (−0.56, 0.72)	0.815	−0.21 (−1.00, 0.50)	0.565

AD, Alzheimer’s disease dementia; APOE, apolipoprotein E.

^a^ Values are represented as medians [25th, 75th].

^b^ The adjusted model (logistic regression model) includes “group” (control and AD) as predictors and age, body mass index (BMI), history of diabetes, history of hypercholesterolemia, and APOE genotype as covariates.

^c^ The adjusted model (logistic regression model) includes “group” (control and AD) as predictors and age, BMI, history of diabetes, and history of hypercholesterolemia as covariates.

The bold values represent statistical significance.

**Figure 3:**
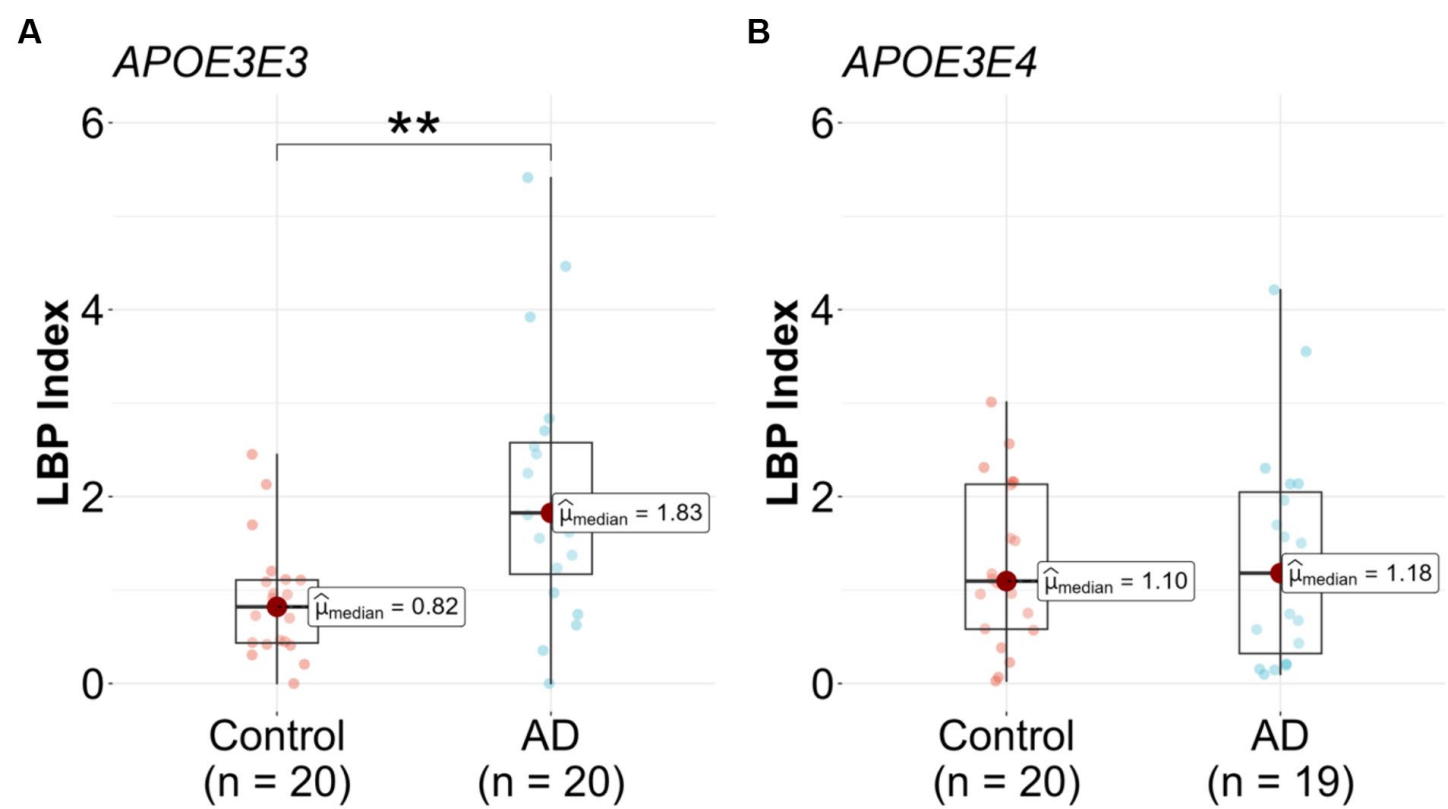
Lipopolysaccharide binding protein (LBP) in Alzheimer’s disease (AD) patients compared to controls stratified by genotype. LBP were measured in *n* = 79 participants, **(A)** *APOE3/E3 n* = 20 control and *n* = 20 AD, and **(B)** *APOE3/E4 n* = 20 control and *n* = 19 AD. Analysis was done in R utilizing logistic regression model.

Upon stratification by *APOE* genotype, a significant association between the LBP index and AD diagnosis was observed in the *APOE3/E3* subgroup. The median LBP index values for the control and AD groups were 0.82 (25th-75th percentile: [0.43, 1.11]) and 1.83 (25th-75th percentile: [1.17, 2.58]), respectively (Figure 3A and Table 2). The unadjusted model exhibited a significant association (*β* = 1.3, 95% CI: [0.47, 2.41], *p* = 0.008), and this association remained significant after adjusting for covariates (*p* = 0.049) (Table 2). In the *APOE3/E4* subgroup no significant associations were found in either the unadjusted (*p* = 0.815) or adjusted models (*p* = 0.561) (Figure 3B and Table 2).

### 2.4 LBP index correlations with cognitive measures

**Figure 4:**
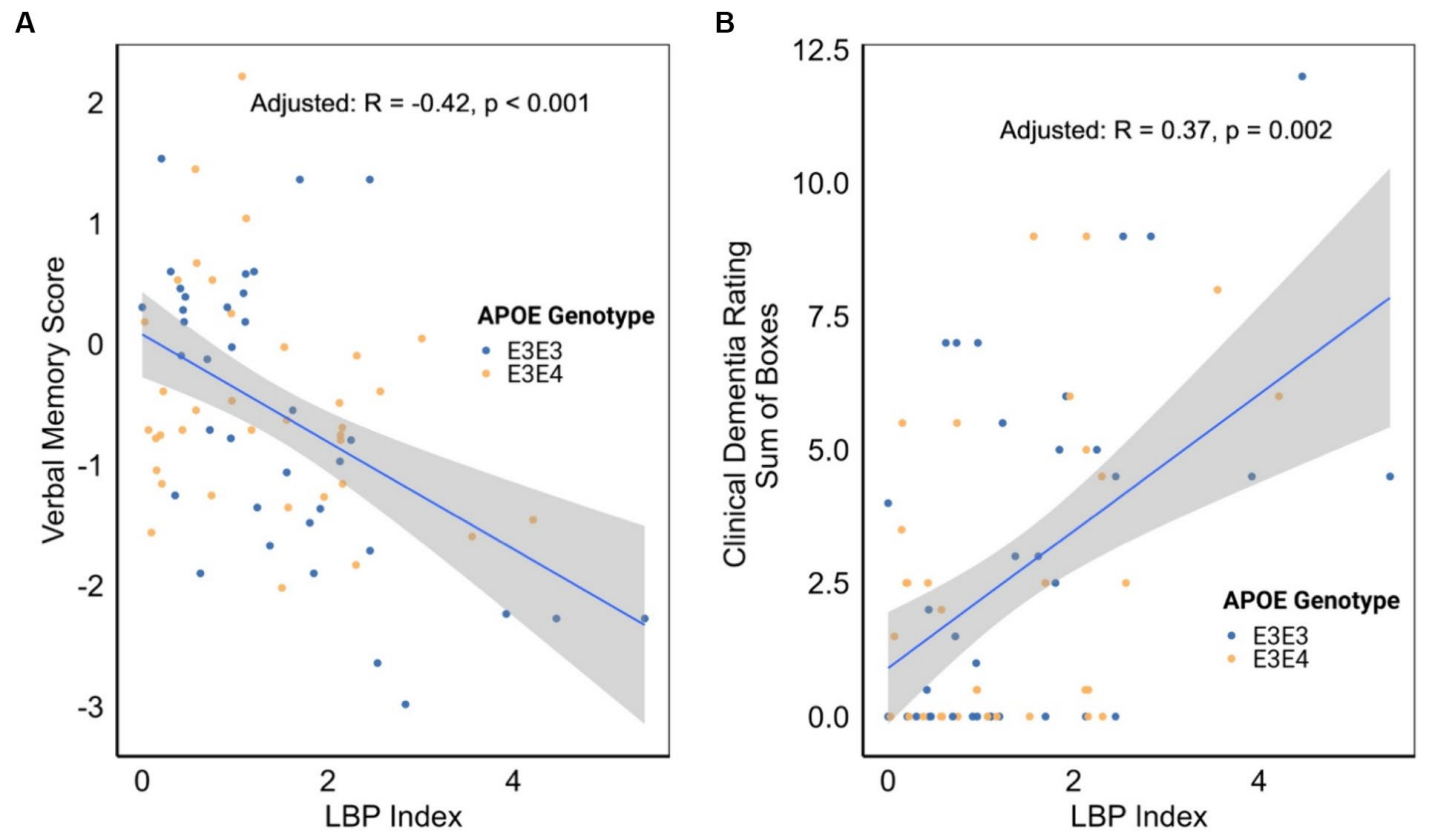
**(A)** Correlation between lipopolysaccharide binding protein (LBP) index and verbal memory score. **(B)** Correlation between LBP index and clinical dementia rating. Correlation analyses were stratified by apolipoprotein E (*APOE*) genotype.

An examination of the associations between the LBP index and various cognitive, functional, and imaging scores, adjusted for *APOE* genotype, revealed significant inverse correlations between the LBP index and verbal memory score (VMS) [*R* = −0.42, 95% CI (−0.60, −0.20), *p* < 0.001, Figure 4A and Table 3]. Furthermore, the LBP index showed a positive correlation with the Clinical Dementia Rating (CDR) sum of boxes [*R* = 0.37, 95% CI (0.14, 0.57), *p* = 0.002, Figure 4B]. These associations persisted after adjusting for the *APOE* genotype, with adjusted correlations remaining statistically significant for both VMS and the CDR sum of boxes. No significant association was observed between the LBP index and white matter hyperintensities volume.

**Table 3:** Correlation analysis adjusting for *APOE* genotype between LBP Index either cognitive, functional, and imaging scores across diagnoses.

Comparison across diagnoses (Control and AD)	Cognitive				Functional	Imaging
Characteristic	Verbal memory score	Executive function score	Semantic memory score	Spatial score	CDR sum of boxes	White matter hyperintensities volume^a^
***N***	72	75	74	68	68	69
LBP index (1.25 Density)						
R	−0.42	−0.21	−0.08	−0.02	0.37	−0.10
95%CI	(−0.60, −0.20)	(−0.42, 0.03)	(−0.31, 0.16)	(−0.27, 0.22)	(0.14, 0.57)	(−0.34, 0.14)
*P* value	**<0.001**	0.076	0.519	0.852	**0.002**	0.399
Adjusted ^b^R	−0.42	−0.20	−0.07	−0.02	0.37	−0.11
Adjusted ^b^95% CI	(−0.60, −0.19)	(−0.42, 0.03)	(−0.29, 0.14)	(−0.27, 0.22)	(0.15, 0.56)	(−0.32, 0.12)
Adjusted ^b^ *p*-value	**<0.001**	0.080	0.529	0.853	**0.002**	0.393

^a^White matter hyperintensities (WMH) volume was normalized to the total intracranial volume. ^b^Adjusted for the APOE genotype.

The bold values represent statistical significance.

## 3 Discussion

LBP is a marker of gut permeability in general, since it correlates strongly with the lactulose/mannitol ratio, the gold standard measure of gut permeability (32), but it is also a specific marker of LPS translocation. Unlike LPS, which is cleared by the system rapidly, LBP remains detectable for up to 24 h after exposure to LPS, making it a particularly suitable marker for studies of patient samples such as those from biorepositories (33). Higher circulating LBP indicates increased endotoxin exposure, which can initiate neuroinflammatory processes implicated in AD (34). LPS has been shown to degrade the blood brain barrier, allowing translocation of endotoxins into the brain and promoting the amyloid beta aggregation and tau tangles characteristic of AD pathophysiology (5, 6, 35, 36). In AD animal models LPS injected peripherally results in neuroinflammation, neuron and memory loss, amyloid beta aggregation and tau hyperphosphorylation (37–41). LPS has also been detected within the parenchyma and blood vessels of the brains of AD patients and has been found to co-localize with amyloid-beta plaques (42, 43). In a previous small study focused on ALS patients, and using 18 AD patients as a control group in addition to age-matched cognitively normal controls, the AD patients had even higher LPS concentrations than ALS patients and both patient groups had higher LPS concentrations than cognitively normal controls (5). In a previous study it was found that baseline LBP independently predicted a 30% higher risk of AD incidence over the course of 12 years (20). However, *APOE* genotype was not measured in this study, therefore the modifying effect of *APOE* genotype on the relationship between LBP and AD risk was previously unknown.

In the current study we measured the concentrations of LBP in the 1.21–1.25 g/mL density fraction of plasma from 39 AD patients and 40 age-matched controls with the two most prevalent *APOE* genotypes *APOE3/E3* and *APOE3/E4*. We found that AD patients overall had higher LBP compared to controls. However, when stratifying by *APOE* genotype we found that the higher LBP was found only in *APOE3/E3* patients but not *APOE3/E4* patients compared to controls. These findings suggest that LBP may be a more important factor in AD pathophysiology in those individuals who are not already at higher genetic risk for AD. This is an intriguing finding as it may point to gut permeability, specifically LPS translocation, as an important factor in AD risk for those individuals who are genetically protected from AD. LBP concentrations were inversely correlated with VMS and positively correlated with CDR. These data suggest that there is a clear link between higher LBP concentrations and lower cognitive function. This is particularly of interest because gut permeability can be reversed through diet and lifestyle interventions in a relatively short period of time. Specifically, higher intake of fruits and vegetables has been linked with lower concentrations of LBP (44). A simple intervention of daily apple consumption significantly decreased LBP concentrations in overweight and obese adults in just 6 weeks (45). The measurement of LBP may be an effective strategy for identifying an actionable AD risk factor in individuals, particularly those who are at genetically low risk of AD (i.e., those with *APOE3/E3* genotype).

Future studies including patients with less prevalent genotypes, especially individuals with the *APOE4/E4* genotype, would be interesting to resolve whether there is a clear *APOE* genotype effect in the relationship between LBP and AD. It is possible that in *APOE4* carriers the genetic risk conferred by *APOE4* is the stronger force in driving AD pathophysiology, whereas gut permeability may be a more critical factor in individuals with the *APOE3/E3* genotype. Future studies are needed to measure LBP in larger cohorts of AD patients and controls, as well as individuals across the spectrum of AD progression and across age and *APOE* genotypes. Future studies are also needed to understand the factors contributing to increased LBP in the context of AD. For example, studies are needed to identify specific gut microbiome compositional features that are associated with higher LBP. It will be important to determine whether it is the presence of beneficial gut microbes known to be involved in supporting gut barrier function, or the absence of pathogenic gut microbes that is more important in increased gut permeability. It will also be important to identify specific dietary factors, patterns, or molecules that can reverse gut permeability.

## 4 Methods and materials

### 4.1 Samples and participants

The detailed study design and the characteristics of the participants involved in this clinical trial have been described previously (21). Briefly, the study involved obtaining plasma samples from 194 participants who were part of the University of California, Davis Alzheimer’s Disease Research Center biorepository. The participants were categorized into three groups including non-demented controls, patients with mild cognitive impairment (MCI), and patients with Alzheimer’s disease dementia (AD). All participants had their *APOE* genotype determined. For this study a subset of *n* = 79 samples out of the 194 were analyzed. The subset was selected randomly based on the following criteria: select *n* = 20 participants with *APOE3/E3* genotype with AD dementia and *n* = 20 without dementia, and *n* = 20 participants with *APOE3/E4* genotype with AD dementia and *n* = 20 without dementia such that the final groups each have equal numbers of male and female participants and are as close as possible to equal average age. After random selection was completed and samples were pulled for analysis, one sample was found to have inadequate sample volume to proceed with further analysis, thus the final sample size for the *APOE3E4* AD dementia group was *n* = 19. Cognitive assessments of the study participants were available and included the Spanish English Neuropsychological Assessment Scales (SENAS), the Clinical Dementia Rating (CDR) scale, and white matter hyperintensities identified through magnetic resonance imaging (21).

### 4.2 HDL isolation

HDL particles were isolated from plasma through a two-step ultracentrifugation process, followed by size exclusion chromatography, as described previously (21, 46). Briefly, a 500 μL aliquot of platelet-free plasma was subjected to ultracentrifugation using a Beckman Optima MAX-TL ultracentrifuge with a fixed angle rotor TLA-110 at 110,000 RPM for 0.5 h using an Optiseal tube and a density cushion of 1.006 g/mL potassium bromide (KBr). This step isolated triglyceride-rich lipoproteins (TRLP, chylomicrons and very low-density lipoprotein (VLDL) particles) lighter than 1.006 g/mL. For the second ultracentrifugation at 110,000 RPM for 3.5 h with a 1.210 g/mL KBr cushion, low density lipoprotein (LDL)/HDL particles denser than 1.006 g/mL but lighter than 1.210 g/mL were collected. The particles of density range 1.006–1.21 g/mL were then injected into a size exclusion chromatography (SEC) column to separate HDL, LDL, and albumin by size. Each fraction underwent buffer exchange using a 3 kDa molecular weight cut-off filter to remove the salt solution.

The remaining plasma protein fraction containing all plasma components >1.21 g/mL in density was then further subjected to an additional ultracentrifugation step to obtain HDL particles within the density range of 1.21–1.25 g/mL. A solution was prepared by mixing 1200 μL of *d* = 1.34 g/mL KBr and HPLC grade water with 2,700 μL of the >1.21 g/mL plasma protein fraction, yielding a total volume of 3,900 μL of *d* = 1.25 g/mL solution. Using 4.7 mL Optiseal tubes, 800 μL of the prepared *d* = 1.25 g/mL KBr solution was underlayed with 3,900 μL of the *d* = 1.25 g/mL density plasma protein solution and topped off with *d* = 1.25 g/mL KBr solution. The samples were ultracentrifuged at 110,000 rpm for 6 h using a Beckman Optima MAX-TL ultracentrifuge with a fixed angle rotor TLA-110. After ultracentrifugation, the top 2 mL of the Optiseal tube, representing the *d* = 1.21–1.25 g/mL fraction was carefully collected. The fraction was then concentrated and buffer exchanged to HPLC grade water by centrifugation at 4500 rpm for 7 min using an Amicon ultrafiltration unit. To ensure uniformity, HPLC grade water was added so that all samples had a final volume of 250 μL, which was then transferred to a storage tube and stored at −80°C for subsequent analysis.

### 4.3 Analysis of LBP in fractions

For the determination of LBP concentrations across all plasma fractions, all of the fractions obtained during the ultracentrifugation/SEC process were collected including the TRLP, LDL, intermediate density lipoprotein (IDL), large (HDL-L), medium (HDL-M), and small (HDL-S) HDL particles at 1.21 g/mL density, albumin (ALB), as well as the dense HDL (*d* = 1.21–1.25 g/mL).

An initial assay was conducted on fractions TRLP, LDL, HDL-L, HDL-M, HDL-S, ALB, and 1.25 g/mL density fraction to determine where LBP was enriched. Given the variability in individual protein concentrations among the samples, the total protein concentration in each sample was optimized to reach concentrations that would fall into the quantifiable range for the Human LBP assay [Abcam LBP assay kit (ab213805)]. The optimal protein concentration range for the assay was determined to be that found in a 1:800 dilution of plasma. The microBCA (micro bicinchoninic acid) protein assay using the Thermo Scientific Micro BCA^™^ Protein Assay Kit (Catalog number: 23235) was used to determine protein concentrations in each fraction. Based on the protein concentration measurements each fraction/sample was diluted to obtain a protein concentration in range of the assay. The samples were then loaded into a 96-well plate, including the internal standards, and the assay was performed using the manufacturer’s protocol. For the further analysis of the 1.21–1.25 g/mL fraction from all *n* = 80 participants, an additional quality control (QC) plasma sample was included on each plate to adjust for inter-plate variability.

### 4.4 Statistical analysis

Statistical analyses were performed using the R programming language, version 4.2.2 (47). To compute the LBP index, LBP measurements were normalized, taking into account the QC values present on each respective plate. Normality of the data was examined through the Shapiro–Wilk test, while the Levene test was employed to ascertain the equality of variances among the groups. Because the LBP index did not meet the conditions for normality and variance homogeneity, the data were described in terms of median values, accompanied by the 25th and 75th percentiles for distribution.

Data visualization, including the creation of figures, was facilitated using the ggstatsplot package (48). In the context of the binary logistic regression model, several covariates were incorporated into the analysis, including age, sex, body mass index (BMI), documented history of diabetes, recorded history of hypercholesterolemia, and the *APOE* genotype. Covariates were included in the regression model if their *p*-value fell below the 0.1 threshold when considering participant characteristics within the combined group. Both unadjusted and adjusted *p*-values were subsequently reported.

To explore possible relationships between cognitive scores (verbal memory, executive function, spatial, semantic memory and clinical dementia rating scale) and the LBP index, partial correlation analyses were conducted, adjusting for the influence of the *APOE* genotype. For the purposes of determining statistical significance, a threshold of *p* < 0.05 was applied.

## 5 Conclusion

The key finding of this study was a significant elevation in LBP concentration within the *d* = 1.21–1.25 g/mL plasma fraction in AD patients compared with age-matched cognitively normal controls. When stratifying for *APOE* genotype, the higher LBP was found only in AD patients with the *APOE3/E3* but not the *APOE3/E4* genotype compared to controls. LBP was negatively correlated with verbal memory and positively correlated with clinical dementia rating, indicating that gut permeability as measured by LBP is linked with lower cognitive function. Together, these results suggest that circulating endotoxin may play an important role in AD development, especially among genetically low-risk individuals.

## Data availability statement

The raw data supporting the conclusions of this article will be made available by the authors, without undue reservation.

## Ethics statement

The studies involving humans were approved by the Institutional Review Board of the University of California at Davis (protocol code: 227656, date of approval: 03/27/2017). The studies were conducted in accordance with the Declaration of Helsinki, local legislation and institutional requirements. The participants provided their written informed consent to participate in this study. Written informed consent was obtained from the individual(s) for the publication of any potentially identifiable images or data included in this article.

## Author contributions

ER: Conceptualization, Methodology, Validation, Formal analysis, Investigation, Writing – original draft preparation, Writing – review & editing. BH: Methodology, Software, Validation, Investigation, Data curation, Writing – review & editing, Visualization. RP: Investigation, Writing – original draft, Writing – review & editing. JA: Validation, Investigation, Writing – review & editing. DH: Investigation, Writing – original draft, Writing – review & editing. IM: Conceptualization, Resources, Writing – review & editing, Funding acquisition. L-WJ: Conceptualization, Resources, Writing – review & editing, Funding acquisition. CL: Conceptualization, Resources, Writing – review & editing. AZ: Conceptualization, Methodology, Validation, Formal analysis, Resources, Supervision, Project administration, Funding acquisition, Writing – original draft, Writing – review & editing.
